# Investigation of the Thermomechanical Response of Cyclically Loaded NiTi Alloys by Means of Temperature Frequency Domain Analyses

**DOI:** 10.3390/ma14247866

**Published:** 2021-12-19

**Authors:** Sofia Di Leonardo, Riccardo Cappello, Gaetano Burriesci, Giuseppe Pitarresi

**Affiliations:** 1 Ri.MED Foundation, Bioengineering Group, Via Bandiera 11, 90133 Palermo, Italy; sofia.dileonardo@unipa.it (S.D.L.); gburriesci@fondazionerimed.com or g.burriesci@ucl.ac.uk (G.B.); 2 Cardiovascular Engineering Laboratory, UCL Mechanical Engineering, University College London, Torrington Place, London WC1E 7JE, UK; 3 Dipartimento di Ingegneria, Università degli Studi di Palermo, Viale delle Scienze Ed. 8, 90128 Palermo, Italy; riccardo.cappello@unipa.it

**Keywords:** shape memory alloys, Nitinol, super-elasticity, Thermoelastic Stress Analysis, Digital Image Correlation, thermomechanical couplings

## Abstract

Nickel–Titanium (NiTi) shape memory alloys subjected to cyclic loading exhibit reversible temperature changes whose modulation is correlated with the applied load. This reveals the presence of reversible thermomechanical heat sources activated by the applied stresses. One such source is the elastocaloric effect, accounting for the latent heat of Austenite–Martensite phase transformation. It is, however, observed that when the amplitude of cyclic loads is not sufficient to activate or further propagate this phase transformation, the material still exhibits a strong cyclic temperature modulation. The present work investigates the thermomechanical behaviour of NiTi under such low-amplitude cyclic loading. This is carried out by analysing the frequency domain content of temperature sampled over a time window. The amplitude and phase of the most significant harmonics are obtained and compared with the theoretical predictions from the first and second-order theories of the Thermoelastic Effect, this being the typical reversible thermomechanical coupling prevailing under elastic straining. A thin strip of NiTi, exhibiting a fully superelastic behaviour at room temperature, was investigated under low-stress amplitude tensile fatigue cycling. Full-field strain and temperature distributions were obtained by means of Digital Image Correlation and IR Thermography. The work shows that the full field maps of amplitude and phase of the first three significant temperature harmonics carry out many qualitative information about the stress and structural state of the material. It is, though, found that the second-order theory of the Thermoelastic Effect is not fully capable of justifying some of the features of the harmonic response, and further work on the specific nature of thermomechanical heat sources is required for a more quantitative interpretation.

## 1. Introduction

Nickel–Titanium, NiTi, is a metallic alloy which allows a tuneable and controlled shape memory effect and superelastic behaviour. This provides some unique features that find application in several industrial fields, such as: thermal and electrical actuators, dampers, biomedical devices, etc. Most of these applications rely on the ability of the material to repeatedly undergo wide reversible straining. Therefore, the fatigue behaviour of NiTi alloys is an important aspect of characterisation for safe design. 

When subject to cyclic loading, Nickel–Titanium (NiTi) shape memory alloys exhibit peculiar temperature modulations that are driven by the applied cyclic loads. This implies the presence of reversible thermomechanical heat sources that are activated by the applied stresses. The most influential of such sources is the elastocaloric effect, which accounts for the latent heat of Austenite⇄Martensite (A⇄M) phase transformation (PT). The A→M and M→A PTs respectively determine a release and an absorption of heat, which warms and cools the material at the site of the transformation. When the range of a cyclic load is wide enough to activate a two-way A⇄M PT at each cycle, the result is a modulation of temperature whose amplitude can reach several degrees Celsius.

When the range of cyclic loading is not sufficient to activate this two-way PT within the cycle, the material still exhibits a strong cyclic temperature modulation. This work, then, proposes an investigation on the nature of thermomechanical couplings in NiTi strips under cyclic low-amplitude tensile loading. In particular, the thermomechanical behaviour is monitored at strain amplitudes which do not activate a cyclic A⇄M PT. Indeed, most devices designed to functionally exploit the superelastic behaviour of NiTi are only occasionally strained up to A⇄M PT activation. An example is given by endovascular prostheses, which are collapsed and expanded during implantation, while their routine fatigue loading is carried out at strain variations that do not involve further A⇄M PT [1]. It is, then, relevant to investigate the NiTi thermomechanical behaviour under such conditions, where the heat source related to the Thermoelastic Effect is expected to become the prominent heat source.

In the last ten years, the investigation of PT and thermomechanical behaviour in superelastic NiTi has significantly advanced thanks to the adoption of full-field techniques for measuring strains and temperature [2,3,4,5,6]. Digital Image Correlation (DIC) and Infrared Thermography (IRT) have proven to be particularly useful in revealing the localised features of PT [7,8,9,10,11]. A significant majority of fatigue studies so far has then focused on A⇄M PT fatigue, i.e., the case when each loading cycle involves a two-way PT [12,13,14]. In these conditions, fatigue strength is primarily related with the ability of the material to cyclically sustain a back-and-forth transition between the Austenite and Martensite states, which may result in a low-cycle fatigue strength. This case, though, is primarily relevant in applications such as solid-state cooling, or other functional applications specifically relying on the maximum recoverable strain evolution [15]. Under such high-strain fatigue, the latent heat of transformation is also the major thermomechanical heat source, and its reversible nature is reflected in the modulation of the temperature signal, which is in phase with the loading signal.

Some authors have proposed modelling the transformations going on in the stress/strain hysteresis loop of a cyclically loaded NiTi sample by means of a thermodynamic energy balance [16,17]. This can be summarised in a local form of the heat diffusion equation which includes heat source terms specific for shape memory alloys, that account for the latent heat of PT [16,18].

Under low-amplitude strain fatigue, assuming adiabatic conditions, neglecting the material intrinsic dissipation and assuming also that no latent heat of transformation is released, the main heat source that remains in this thermodynamic energy balance is associated with the Thermoelastic Effect. This term of the diffusion equation would allow linking the temperature changes with stresses/strains, through the first- or second-order laws of the Thermoelastic Effect. Only a few works have explored the validity and utility of the previous assumptions [19,20]. A noteworthy outcome of these studies was that the temperature appears to change in phase with the load, against the predictions of the first order Thermoelastic theory. Similar in-phase responses are observed and reported by others [2,21], generally observing a quasi-linear and spatially homogeneous increase of temperature in the sample immediately after the start of tensile loading, up to the A⇄M PT. A few works have also reported a decrease of temperature at the very start of the tensile test, which soon reverses to an increase as the load increases, but well before the initiation of the A→M plateau [22]. By adopting a higher-order thermoelastic theory, it has been found that a positive-valued derivative of the Young’s modulus with temperature could justify a thermoelastic signal in phase with the external load [19,20]. A relatively high and positive *dE/dT* was indeed measured experimentally, both in the Austenite and Martensite states, at near room temperature. It has also been observed that some Nitinol grades might activate an intermediate stare between Austenite and Martensite, consisting of an R-type PT [23]. A stress-activated R-type PT also determines a change in the Young’s modulus *E*, which might have an impact on the thermoelastic signal and also on the second harmonic.

The present work investigates the behaviour of a NiTi strip under a cyclic tensile load applied under strain control, with a positive mean strain value and an amplitude smaller than the difference between the two A⇄M transformation plateau stresses. Such a loading condition will generally correspond to a stationary distribution of Austenite or Martensite zones, whose extent depends on the value of the mean strain [24]. Both DIC and IRT are applied. DIC makes it possible to control the phase distribution during the fatigue loading, while IRT is used to sample the temperature signal during cycling. Since the sampled temperature shows a similar modulation of the applied load, and hence a marked reversible nature, a harmonic analysis is proposed, carried out using Discrete Fourier Transform signal processing.

The proposed investigation of the frequency content and the harmonic features of the temperature signal has the specific purpose of investigating the nature of the heat source feeding such temperature variation. The paper presents an analysis of the first and second harmonics of temperature (i.e., the temperature changes at the load frequency and twice the load frequency), with the purpose of establishing whether the temperature modulation behaviour is fully predicted by the known Thermoelastic Effect theories.

## 2. NiTi Thermomechanical Modelling

Several authors have modelled the NiTi thermomechanical behaviour within the assumptions and the theory of thermodynamics of irreversible processes for solid systems [16,17,25]. Under quasistatic conditions, the only work done on a solid system can be assumed to be stored as strain energy. Therefore, the local thermodynamic status of the system is determined by a set of state variables: the temperature *T*, the strain tensor *ε_ij_* and other internal state variables. For the case of shape memory alloys, an internal state variable is needed to describe the micro lattice phase status and its changes. Other variables may also be added to describe damage onset. Therefore, all internal variables can be included into a vector **x**. 

By combining the first and second principles of thermodynamics, introducing the Helmholtz free energy thermodynamic potential, *H*, it is possible to obtain the local energy balance in the form of a heat conduction law, as follows [16,26]:

(1) $$\rho C_{\varepsilon }\dot{T}-k\nabla^{2}T=D_{m}+T\frac{\partial \sigma_{ij}}{\partial T}{\dot{\varepsilon }}_{ij}^{e}+T\rho \frac{\partial^{2}H}{\partial T\partial x}\dot{x}+q_{s}$$

where *ρ*, *C_ε_* and *k* describe the material density, specific heat at constant volume and heat conductivity, *D_m_* is the intrinsic dissipation and *q_s_* the heat exchange between the body and the surroundings. 

When the material undergoes cyclic loading over a sufficiently high frequency threshold, it can be assumed that the transformation is adiabatic, i.e., no internal heat conduction arises and the second term on the left-hand side of Equation (1) is neglected. If the loading amplitude is sufficiently small to be included within a purely elastic material response, insufficient to activate A⇄M PT, then it is possible to assume that $\dot{x}=0$ . Moreover, the internal mechanical dissipation can also be assumed to be null and no other internal heat sources are present. Under these assumptions Equation (1) is only left with the thermoelastic coupling term, and becomes [27]:

(2) $$\dot{T}=\frac{T}{\rho C_{\varepsilon }}\frac{\partial \sigma_{ij}}{\partial T}{\dot{\varepsilon }}_{ij}^{e}$$

Integrating over a finite time, Equation (2) can also be written as:

(3) $$\Delta T=\frac{T_{o}}{\rho C_{\varepsilon }}\frac{\partial \sigma_{ij}}{\partial T}\Delta \varepsilon_{ij}$$

### 2.1. Thermoelastic Coupling

The conditions leading to Equation (3) make it possible to establish a stress analysis technique, generally referred to as Thermoelastic Stress Analysis (TSA) [19], by means of which the temperature harmonic content is filtered from the temperature signal and correlated with stress metrics for structural evaluations. 

Further development of Equation (3) requires the evaluation of the derivative of the stress tensor with T. Assuming the generalised Hooke’s elastic law, two different Thermoelastic laws are derived [27].

The first order thermoelastic law is obtained by assuming that all elastic constants and the coefficient of thermal expansion have a negligible dependence on temperature changes. This holds true for most materials around room temperature, and Equation (3) can be further simplified to become a linear correlation between temperature change and the change of the strain or stress first invariant:

(4) $$\Delta T=-T_{o}\frac{\alpha }{\rho C_{\varepsilon }}\frac{E}{\left(1-2\nu \right)}\Delta \varepsilon_{ii}=-T_{o}\frac{\alpha }{\rho C_{p}}\Delta \sigma_{ii}$$

The correlation with the first strain invariant also suggests that the thermoelastic coupling term is related with the local elastic reversible volume change. This is often identified as the Thermoelastic Effect. 

A second-order thermoelastic law was derived by Wong et al. [28,29], where the dependence of elastic parameters from T is not neglected. This leads to a more complex stress metric that can be summarised as:

(5) $$\rho C_{\varepsilon }\frac{\dot{T}}{T}=-\left[\alpha +\left(\frac{\nu }{E^{2}}\frac{\partial E}{\partial T}-\frac{1}{E}\frac{\partial \nu }{\partial T}\right)\sigma_{I}\right]{\dot{\sigma }}_{I}+\left[\frac{\left(1+\nu \right)}{E^{2}}\frac{\partial E}{\partial T}-\frac{1}{E}\frac{\partial \nu }{\partial T}\right]\sigma_{i}{\dot{\sigma }}_{i}$$

where *σ_I_* = *σ*_1_ + *σ*_2_ + *σ*_3_ is the first stress invariant and *σ_I_* = *σ*_1_, *σ*_2_, *σ*_3_ are the principal stress components. If a one-dimensional stress field is considered where *σ_I_* = *σ*_1_ + *σ*_2_ + *σ*_3_, *σ*_1_ = *σ* and *σ*_2_ = *σ*_3_ = 0, then Equation (5) simplifies in:

(6) $$\rho C_{\varepsilon }\frac{\dot{T}}{T}=-\left[\alpha -\frac{1}{E^{2}}\frac{\partial E}{\partial T}\sigma \right]\dot{\sigma }$$

To achieve adiabatic conditions, and to improve the signal to noise ratio, TSA usually applies cyclic loads [30,31]. Let us consider the case of a tensile load varying as a sin wave with a frequency *ω* i.e., *σ* = *σ_m_* + Δ*σ* sin(*ωt* + *ϕ*), and an initial phase *ϕ* = 0, then Equations (2) and (6) become:

(7) $$\Delta T=-T_{o}K_{o}\Delta \sigma \sin\left(\omega t\right)=\left|T_{\omega ,1}\right|F_{1}$$

(8) $$\Delta T=-T_{o}\left(K_{0}-K_{1}\sigma_{m}\right)\Delta \sigma \sin(\omega t)-T_{o}K_{2}{\left(\Delta \sigma \right)}^{2}\cos(2\omega t)=\left|T_{\omega ,2}\right|F_{2}+\left|T_{2\omega ,2}\right|S_{2}$$

where:

$$\begin{array}{l} K_{0}=\frac{\alpha }{\rho C_{\varepsilon }};K_{1}=\frac{1}{\rho C_{\varepsilon }}\frac{1}{E^{2}}\frac{\partial E}{\partial T};K_{2}=\frac{1}{\rho C_{\varepsilon }}\frac{1}{4E^{2}}\frac{\partial E}{\partial T}=\frac{K_{1}}{4}\\ T_{\omega ,1}=T_{o}K_{o}\Delta \sigma ;T_{\omega ,2}=T_{o}\left(K_{o}-K_{1}\sigma_{m}\right)\Delta \sigma ;T_{2\omega ,2}=T_{o}K_{2}{\left(\Delta \sigma \right)}^{2}\\ F_{1}=-\sin\left(\omega t\right);F_{2}=\sin\left(\omega t\right);S_{2}=-\cos\left(2\omega t\right); \end{array}$$

where *F* and *S* are the symbols used here for the time varying part of each harmonic term. *F* is related to the “first harmonic”, i.e., the harmonic at the frequency *ω*, and *S* is related to the “second harmonic”, at the frequency 2*ω*. Subscripts in *F* and *S* identify the Thermoelastic Effect formulation, 1 for first order and 2 for second order. 

Only a few works have investigated the thermoelastic effect in shape memory alloys [19,20,23]. Cycling under stable Austenite or Martensite phase revealed that the temperature harmonic at the loading frequency, *ω*, is in phase with the applied load. This is also confirmed by most quasistatic tests shown in the literature, which generally report a tendency of the material to heat up when the traction load increases and cool down when the traction load is reversed, in both fully Austenite and fully Martensite stages. This behaviour can be justified assuming that the material follows a second-order thermoelastic law, where the sign of the first harmonic in Equation (8) (i.e., the temperature change at *ω*) is positive, due to becoming *K*_1_*σ_m_ > Ko* [19]. This holds true when the value of *dE/dT* satisfies:

(9) $$\frac{\partial E}{\partial T}>\frac{\alpha E^{2}}{\sigma_{m}}$$

Summarising:

1. If *dE/dT* < 0 → *F*_2_ = −sin(*ωt*) and *S*_2.1_ = cos(2*ωt*)
2. If *dE/dT* > 0 but *dE/dT* < αE_2_/*σ_m_* → *F*_2_ = −sin(*ωt*) and *S*_2.2_ = −cos(2*ωt*)
3. If *dE/dT* > αE_2_/*σ_m_* → *F*_2_ = sin(*ωt*) and *S*_2.2_ = −cos(2*ωt*).

While the above condition (1) is usually followed by most common metals and alloys, the experimental evidence for NiTi is that the temperature harmonic at the frequency *ω* is in phase with the load and therefore only the third condition is compliant with the observed material behavior, i.e., *F*_2_ = sin(*ωt*) and *S*_2_ = −cos(2*ωt*).

For typical material properties of NiTi alloys, which for the initial fully Austenite stage are α = 11 × 10^−6^ °C^−1^ and *E* around 40 GPa, considering a mean stress *σ_m_* between 10 and 400 MPa, to satisfy the inequality in Equation (9), *dE/dT* should be higher than 1760–44 MPa/°C. This threshold is commonly exceeded in reported tests (e.g., in [19] it is reported a measured value of *dE/dT* = 2231 MPa/°C, obtained at room temperature *T_o_* = 25 °C), with *dE/dT* becoming significantly higher when *σ_m_* approaches the upper A→M plateau. For a mean stress of 150 MPa and *dE/dT* ≈ 2000 MPa/°C, consistent with reported data [19], the following coefficients arise in Equation (8):

(10) $$\rho C_{\varepsilon }\frac{\Delta T}{T_{o}}\approx 7.75\times 10^{-6}\Delta \sigma \sin(\omega t)-0.31\times 10^{-6}{\left(\Delta \sigma \right)}^{2}\cos(2\omega t)$$

where the coefficient of the second harmonic is about an order of magnitude smaller than that of the first harmonic, but proportional to the square of the stress amplitude. Hence, a value of Δ*σ* > 7.75/0.31 = 25 MPa would result in a second harmonic amplitude higher than the first harmonic. Therefore, the nonlinear thermoelastic effect at 2*ω* can become significant, contrary to more conventional low-strength ductile materials, where the 2*ω* component due to the thermoelastic effect is negligible and the observed second harmonic is often governed by intrinsic dissipation [32].

### 2.2. Validation of the Thermomechanical Model

As outlined above, the principal thermomechanical source activated during a low-amplitude fatigue cycling can be reduced to the Thermoelastic Effect, which is compatible with a reversible temperature variation, as is observed from testing. Previous works investigating the nature of the Thermoelastic Effect in NiTi have focused only on retrieving and analysing the first harmonic, i.e., the temperature harmonic at the loading frequency *ω*. The previous analytical model suggests that the thermoelastic effect is expected to produce a meaningful signature also in the second harmonic 2*ω*. Therefore, the experimental evaluation proposed in the next sections is mainly aimed at analysing the harmonic content of the temperature signal, and to verify its compliance with the previous analytical framework and if it can be used to extract further information on the thermomechanical response of the material.

The different predictions in terms of temporal shifts of the first and second temperature harmonic response can be summarised and identified as follows:

- *F*_1_: thermoelastic response at *ω* according to the first order theory (see Equation (7)).
- *F*_2_: thermoelastic response at *ω* according to the second-order theory (Equation (8)) when Equation (9) is satisfied.
- *S*_2.1_: thermoelastic response at 2*ω* according to the second-order theory (Equation (8)) when *dE/dT* < 0.
- *S*_2.2_: thermoelastic response at 2*ω* according to the second-order theory (Equation (8)) when Equation (9) is satisfied.

## 3. Materials and Methods

**Figure 1:**
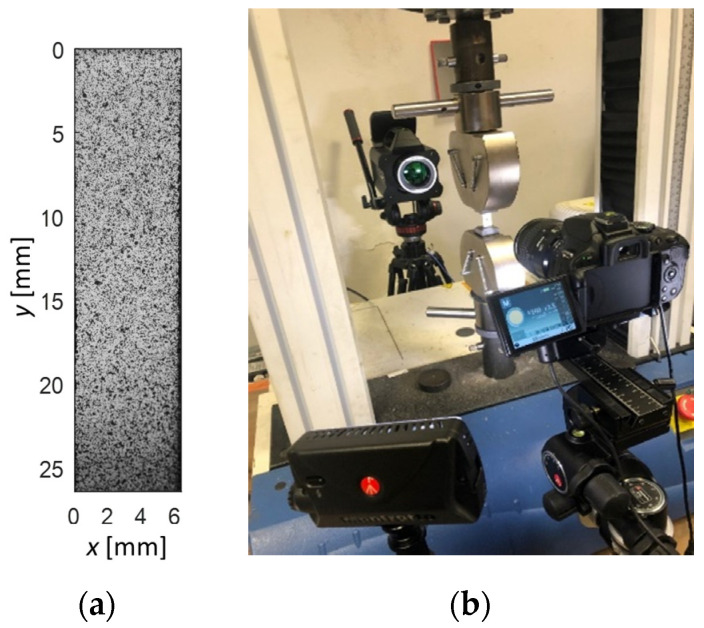
(**a**) Image of tensile strip sample gauge area with the speckled surface for DIC analysis; (**b**) image of tensile testing setup, with the photo camera and the IR camera staring onto the sample faces during the test.

A rectangular strip of NiTi obtained by means of cold rolling, of width equal to 6 mm, gauge length equal to 35 mm and wall thickness equal to 0.2 mm (see Figure 1), was tested under quasi-static and cyclic loading.

During loading, the strain field and temperature were synchronously monitored by 2D Digital Image Correlation from one face of the sample, and Infrared Thermography on the opposite face of the sample. 

Loading was applied on an electro-mechanic Instron 3367 testing machine. Quasi-static loading was applied in displacement control, at a speed of 0.5 mm/min. Cyclic loading was applied by setting a triangular wave shape in the test profiler function of the Instron BlueHill v2.0 software.

### 3.1. Cyclic Loading and Harmonics Analysis

In the present work, cyclic loading is applied by setting a triangular wave modulation of the testing machine crosshead displacement. To evaluate the harmonic content introduced by such loading modulation, the Fourier series of a triangular wave is recalled, considering the time origin at midway of the growing ramp:

(11) $$f\left(x\right)=\frac{8}{\pi^{2}}A\sum_{n=1}^{\infty }\frac{{\left(-1\right)}^{n-1}}{{\left(2n-1\right)}^{2}}\sin\left[\left(2n-1\right)\frac{2\pi }{T}x\right]$$

where *T* is here the fundamental period of the wave and *A* its amplitude.

To better process the experimental data, it is useful to shift the time origin by −90°, i.e., *x* = *t* + *T*/4. Then, the previous relation becomes:

(12) $$f\left(t\right)=-\frac{8}{\pi^{2}}A\sum_{n=1}^{\infty }\frac{1}{{\left(2n-1\right)}^{2}}\cos\left(\left(2n-1\right)\frac{2\pi }{T}t\right)=-\frac{8}{\pi^{2}}A\cos\left(\frac{2\pi }{T}t\right)-\frac{8}{9\pi^{2}}A\cos\left(3\frac{2\pi }{T}t\right)-\frac{8}{25\pi^{2}}A\cos\left(5\frac{2\pi }{T}t\right)-\ldots$$

**Figure 2:**
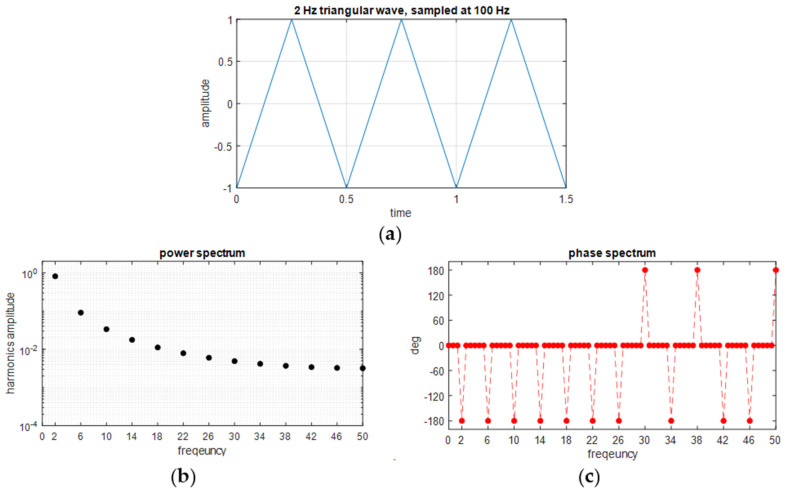
Triangular wave modulation of the input loading: (**a**) triangular wave; (**b**) harmonic amplitude spectrum; (**c**) harmonic phase spectrum. In this example, the spectra are computed by DFT, and the frequency content is shown between 0 and 50 Hz.

The harmonics of the above series expansion can also be approximated by computing the Discrete Fourier Transform (DFT). This is shown in Figure 2, where the DFT is computed using the *fft* function of MATLAB on a chunk of triangular wave containing an integer number of fundamental periods, to minimise spectral leakage [31]. 

From the DFT it is possible to identify the harmonics composing the signal, which are all odd multiples of the fundamental frequency, *ω*. It is also observed that, based on the chosen initial instant of sampling, all harmonics have a 180° phase shift. This value oscillates between ±180° due to numerical approximations.

### 3.2. Thermoelastic Source and Phase Modulation

A triangular wave was preferred for this study, as it produces a harmonic spectrum which comprises a fundamental loading harmonic at *ω*, as well as higher harmonic terms at odd multiple values of *ω*, i.e., 3, 5, 7, … (2*n* + 1)*ω*, with gradually decreasing amplitude. Therefore, the response of the temperature signal at a frequency 2*ω* is expected to be due to the second-order thermoelastic effect.

**Figure 3:**
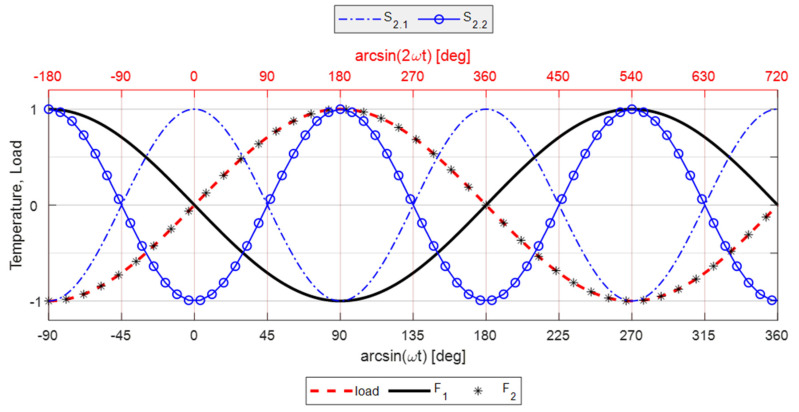
Schematic representation of the angular shifts between different harmonics: load fundamental harmonic *ω*, thermoelastic harmonic *F*_1_, thermoelastic harmonics *F*_2_ and second harmonic terms *S*_2.1_ and *S*_2.2_.

As outlined in Section 2.1, temperature harmonics at *ω* and 2*ω* can assume different characteristic phase shifts compared to the loading wave. Figure 3 provides a schematic representation of such phase differences. In this scheme, the amplitudes of all harmonics were normalised to one, since only the phase difference is being evidenced. Furthermore, the initial time is chosen in accordance with the representation of the loading triangular wave reported in Figure 2. The acquired temperature, load and displacement signals were also truncated so that each acquired signal started at a minimum and contained an even number of periods (in accordance with Figure 2a). This allows comparing the phase of the first and second harmonic of temperature with the predictions sketched in Figure 3.

In the present study the MATLAB *fft* function is used to compute the DFT of the acquired temperature signal during fatigue loading. The harmonics *H_i_* computed by the *fft* algorithm are trigonometric terms of the type:

(13) $$H_{i}=A_{i}\cos\left(2\pi f\cdot t+\phi_{i}\right)$$

For Equation (13) to reproduce the harmonics of Figure 3, it should then be:

Displacement/Load signal → *ϕ* = −180°.

*F*_1_: Thermoelastic first harmonic (first-order formulation or second-order and Equation (9) not satisfied) → *ϕ* = 0°.

*F*_2_: Thermoelastic first harmonic (second-order formulation, Equations (8) and (9)) → *ϕ* = −180°.

*S*_2.1_: Thermoelastic second harmonic (second-order formulation, Equation (8) and *dE/dT* < 0) → *ϕ* = −180°.

*S*_2.2_: Thermoelastic second harmonic (second-order formulation, Equations (8) and (9)) → *ϕ* = 0°.

### 3.3. Plan of Experiments

In this work three different loading histories are applied to the same sample. Table 1 reports the nomenclature of these tests, performed in sequence from the QS to the C_up and C_down. 

**Figure 4:**
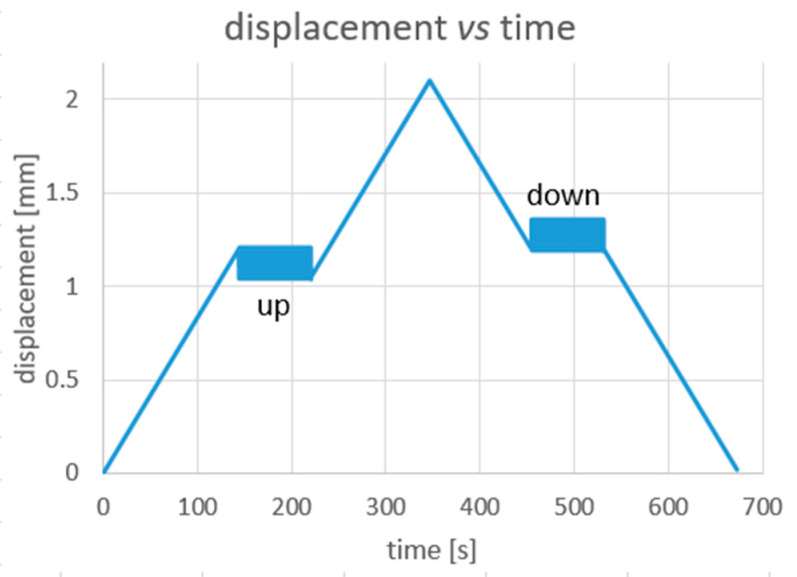
Set displacement vs. time profile for the C test.

**Figure 5:**
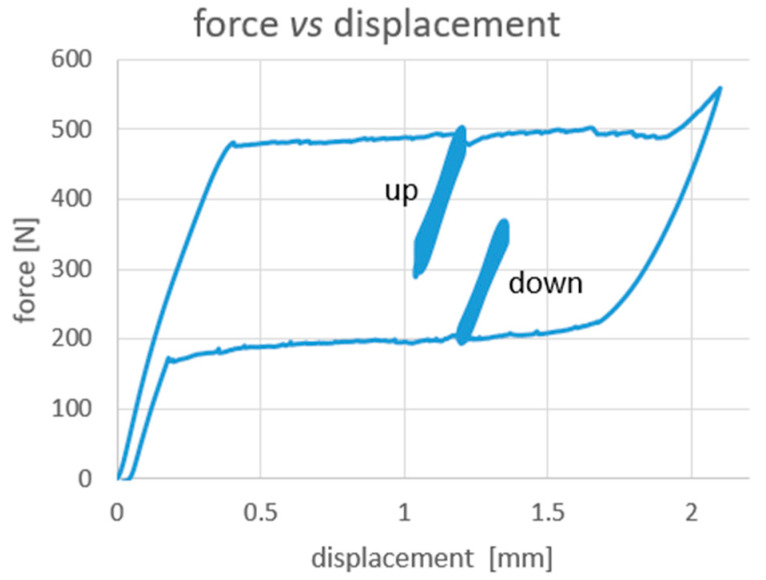
C test measured load vs. displacement curve.

In particular, the load profiles are defined as follows (see also Figure 4 and Figure 5):

- QS:
  1. preloading to 20 N;
  2. ramp from 20 N to 2.4 mm @ 0.5 mm/min;
  3. ramp from 2.4 mm down to 20 N@ 0.5 mm/min;
- C_up and C_down:
  1. ramp from 0 N up to 1.2 mm @ 0.5 mm/min;
  2. C_up: cycling between 1.2 and 1.05 mm @ 0.6 mm/sec for 150 cycles (corresponding to a frequency of the triangular wave of 2 Hz);
  3. ramp from 1.2 up to 2.1 mm @ 0.5 mm/min;
  4. ramp from 2.1 down to 1.2 mm @ 0.5 mm/min;
  5. C_down: cycling between 1.2 and 1.35 @ 0.6 mm/sec for 150 cycles (2 Hz);
  6. ramp from 1.2 mm down to 0.02 mm @ 0.5 mm/min (see also Figure 4).

The values of load and displacement used to define the C test were chosen based on the results of the previous QS test. This was to make sure that the two cycling stages (up and down) were initiated at a longitudinal average strain placed near the mid upper and lower transformation plateaus. This made it possible to test the sample in presence of both Austenite and Martensite regions [22,24]. Additionally, the range of the displacement cycles was chosen so as to avoid any further propagation of the A⇄M PT front during the two cycling windows. A correspondence between the set load and displacement values and the average stress and strain values in the tested sample is reported and commented in Section 4.1.

Finally, all tests were performed at room temperature, with initial temperatures within the range 20–23 °C.

### 3.4. DIC Setup

A 2D Digital Image Correlation setup was implemented to monitor the evolution of the strain field. The setup used a reflex photo camera Nikon D5100 equipped with a Macro lens Nikkor AF-S 105 mm Micro f/2.8, a focus rail slider, and a set of LED panels for optimal scene illumination. The sample was painted with a uniform matt white background and random black speckles applied by means of an airbrush gun. Figure 1a shows the speckled specimen surface. Image sampling was controlled via PC by the software Digicam Control, which allowed a max sampling of 1 picture every 2 s with the time-lapse capture mode. Image correlation was performed using the opens-source software NCORR, operating in MATLAB [33]. The strain maps presented in this paper were obtained by setting the following correlation parameters: strain radius ranging between 12–20 pixels, subset spacing ranging between 4–8 pixels, strain radius 2–4 subset spacing nodes, while the magnification factor ranged between 0.01–0.016 mm/pixel.

### 3.5. Infrared Thermography Setup

The temperature field was measured by means of an Infrared camera staring at the face of the sample opposite that stared by the photo-camera (notice that all IR images reported in Section 4 were flipped left to right to be directly comparable with DIC strain maps). The sample surface was previously painted with a matt black paint to enhance emissivity and reduce reflection. A cooled sensor FLIR X6540sc thermal camera was employed, which allows high thermal resolution and high frame rates (the integration time set in all thermal measurements in this work was 1500 μs). Quasi-static tests were monitored at a frame rate of 1 Hz, while cyclic loadings were sampled at 105 Hz. Data were then pre-processed in Flir Research IR v3.4 and then exported as .mat files for post-processing in MATLAB. 

The temperature sequences acquired during cyclic loading were analysed in MATLAB by applying a Discrete Fourier Transform (*fft*)-based analysis. 

The data processing implemented to evaluate the amplitude and phase of the *ω* and 2*ω* harmonics can be summarised in the following steps [31].

- A time window is selected such to include approximately an integer number of displacement/force and thermogram cycles, starting from a minimum value (or through).
- The truncated data series are then imported into MATLAB. The displacement/force/temperature vs. time curves are least square fitted to a three harmonics equation:
(14) $$Y(x)=Y_{mean}+A_{1}\sin(\omega \cdot x+\phi )+A_{2}\sin(2\omega \cdot x+\phi_{2})+A_{3}\sin(3\omega \cdot x+\phi_{3})$$
  where the unknowns are *Y_mean_*, *A*_1,2,3_ and *ϕ*_1,2,3_. The initial fitting is made with an initial guess of *ω* that is taken as the main peak of a first DFT spectrum. Then, the optimum *ω* is obtained by solving the least square fitting by an iterative optimisation procedure using the MATLAB *patternsearch* function.
- The previous step yields the optimum value of *ω* that is then used to optimise the number of frames of the DFT such that a frequency bin is obtained at the value of *ω*. The lack of spectral leakage in the final DFT spectrum, at *ω*, 2*ω*, 3*ω* and other harmonic peaks, indicates that the optimisation procedure has been successful and the optimal *ω* has been retrieved.

## 4. Results

### 4.1. Quasistatic Loading Test

**Figure 6:**
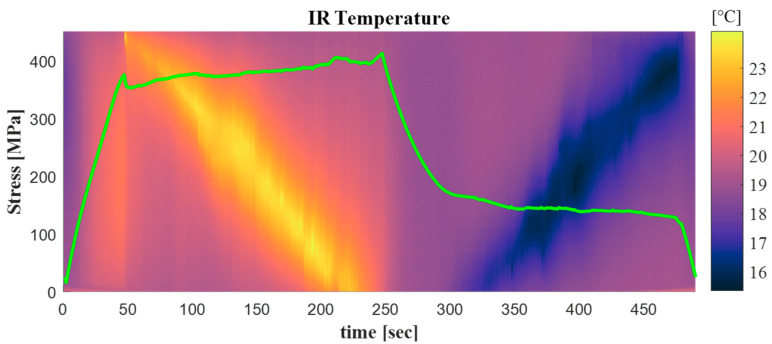
Stress vs. time plots overlapped over the temperature along the centre y-axis vs. time map.

**Figure 7:**
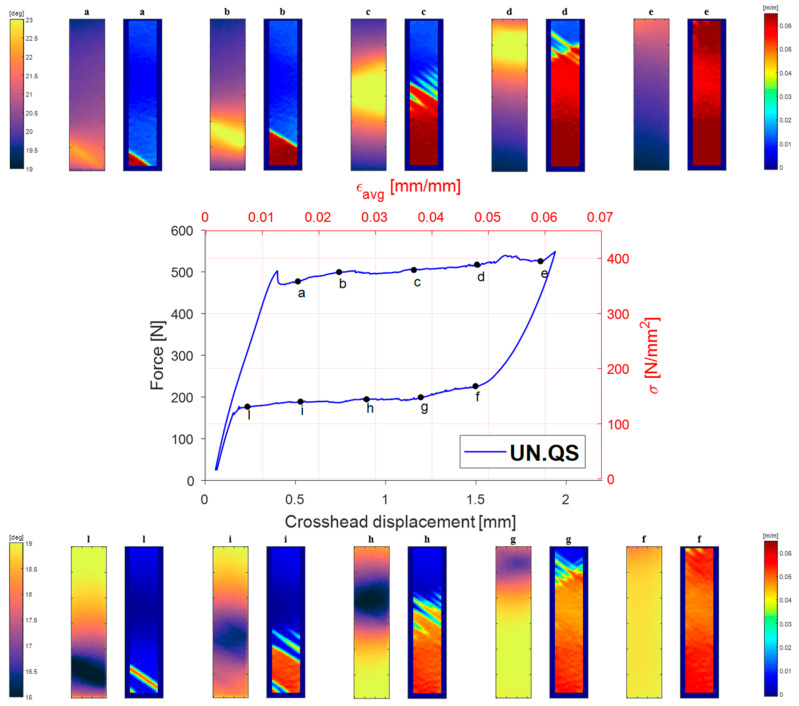
Middle row: plot of the load vs. displacement or engineering stress/strain curves; Top row: thermal and *ε_yy_* DIC maps on selected points along the upper plateau of PT transitory; Bottom row: thermal and *ε_yy_* DIC maps on swelected points along the lower plateau of PT transitory.

Figure 6 and Figure 7 summarise the outcomes from the QS tensile test. It can be observed that the load–displacement curve shows the typical superelastic closed loop evolution exhibited by a NiTi alloy when stressed from a stable Austenite phase. Therefore, a fully superelastic behaviour is detected at room temperature.

In Figure 6, the temperature measured along a vertical line crossing the sample at its mid-width is overlapped with the nominal stress vs. time evolution. From this figure it can be observed that the sample undergoes a uniform temperature increase before reaching the upper plateau, and a uniform decrease when unloaded from the upper to the lower plateaus. 

The plot in Figure 7 also reports a nominal stress vs. nominal strain scale. The length *L_o_* between the upper and the lower grip was 35 mm. This length was used to calculate a global engineering strain, as *ε* = Δ*L/L_o_*, where Δ*L* was given by the crosshead displacement. It is here evidenced that such strain represents only an average strain that does not consider the non-uniform progression of PT, and hence the related non-uniform strain distribution. Therefore, in NiTi samples in general, average stress/strain curves cannot be used to derive an intrinsic material response, as the measured output depends on the extent of the gauge length, regardless of this being monitored with an extensometer or by the machine actuator displacement. 

The two pseudo-plateaus in Figure 7 indicate the overall displacement accumulating during the A→M (upper plateau) and M→A (lower plateau) PTs over the whole sample. Figure 7 also shows some thermograms and *ε_yy_* maps from DIC, with reference to specific points of the curve. From the DIC maps it can be observed that the transformation started from the gripped area, probably because the local stress concentrations favour the onset of PT. In the case of a slow, quasi-static, displacement rate, the PT generally progresses with a unique front advancing in a continuous manner, swiping all the remaining sample area from one grip to the other. This behaviour is observed to be rate dependent, and as strain rate increases, the material tends to form several randomly distributed PT bands. Furthermore, such PT fronts always remain with the same inclination angle, giving rise to the so-called Lüders bands. Next to each DIC map in Figure 7, the temperature map is also provided. Since this was a quasi-static test, the temperature changes, originating from the localised latent heat of transformation, tend to diffuse away by conduction, and then the thermal fronts appear more blurred. Even so, it can be clearly observed that the zone of actual PT is also the zone with higher temperature (in the case of A→M transformation) or lower temperature (in the case of M→A transformation). 

In general, the DIC maps in Figure 7 demonstrate how this full-field technique is able to map the A or M phase zones at a given loading time. This is exploit in the next sections, to analyse the thermal response from each phase.

### 4.2. Cyclic No A⇄M PT—Low-Amplitude Fatigue

**Figure 8:**
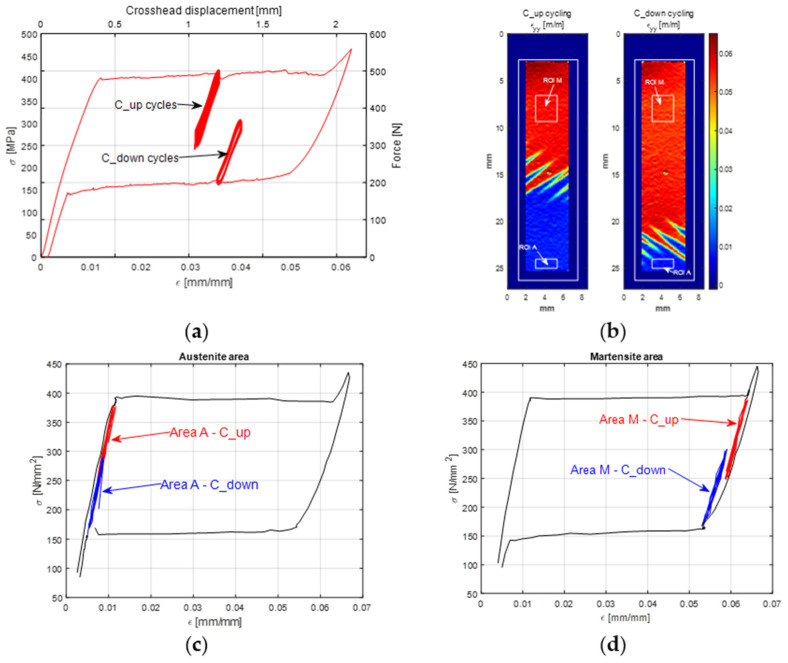
(**a**) Position of the C_up and C_down cyclic stages in the average *σ*-*ε* curve; (**b**) maps of *ε_yy_* from DIC during the cycling stages and Austenite and Martensite regions, which were averaged for the DFT analysis; (**c**,**d**) average *σ-ε* curves from the fully Austenite and Martensite regions, highlighting in red and blue the C_up and C_down cycling stages, respectively.

Figure 8a shows the whole load vs. crosshead displacement curve during the C test, which included the C_up and C_down cyclic windows. In particular, the first cyclic stage started when the sample gauge area had about half of its surface transformed from Austenite to Martensite, as shown by the DIC map in Figure 8b. The amplitude of the first cyclic loading stage was low enough to avoid further A→M PT, and at the same time, the axial stress remained high enough to avoid the activation of any M→A PT. 

A similar behaviour is obtained for the second cyclic loading stage, C_down, this time starting from the lower plateau. Therefore, PTs in both cyclic loadings remained stable. 

The DIC maps in Figure 8b show the distribution of Austenite and Martensite zones. The rectangular regions of interest, ROIs, A and M, were taken inside the Austenite and Martensite zones for monitoring the average temperature and perform a DFT of this average signal. Figure 8c,d show the averaged strain signal from the areas A and M during the C_up and C_down stages. It is interesting to point out that the local values of *σ*-*ε* during cycles are not located at the mid-plateau strain, but are aligned with the Austenite or Martensite elastic loading lines.

**Figure 9:**
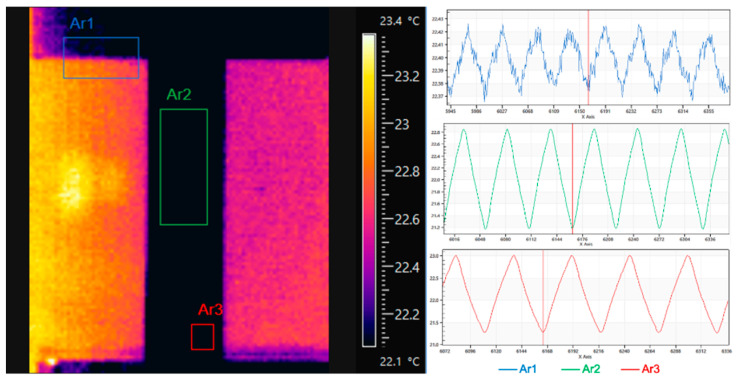
Plots of temperature (**left**) vs. frame sampling from different areas of the specimen and the loading grip (**right**).

The relatively low loading frequency of 2 Hz might prompt the question as to whether adiabatic conditions were effectively onset, as they are required for the simplification of Equation (1) and to obtain a thermoelastic response in phase with the external displacement/load modulation. To check the phase alignment between the applied displacement wave and the thermal output wave, a simple but effective comparison was made between the average temperature from different areas as shown in Figure 9.

Here the comparison is performed in the FLIR software Research IR v.4.0, and the area marked as Ar1 is chosen so as to include part of the upper moving grip and part of the hotter background. In this way, the average temperature from Ar1 is a wave that is directly correlated with the movement of the grip, i.e., to the applied displacement. The other two areas, Ar2 and Ar3, are instead taken from different areas inside the specimen. The vertical red bar appearing in each of the three temperatures vs. time plots indicates a common instant in time sampling, and it is clearly seen that all three curves are well synchronised, i.e., the minimum of the displacement related signal is obtained at a minimum in the temperature curves from Ar2 and Ar3. This is then a cross-check that the loading and temperature waves are in phase with each other.

#### 4.2.1. C_Up—Upper Plateau Cycling 

**Figure 10:**
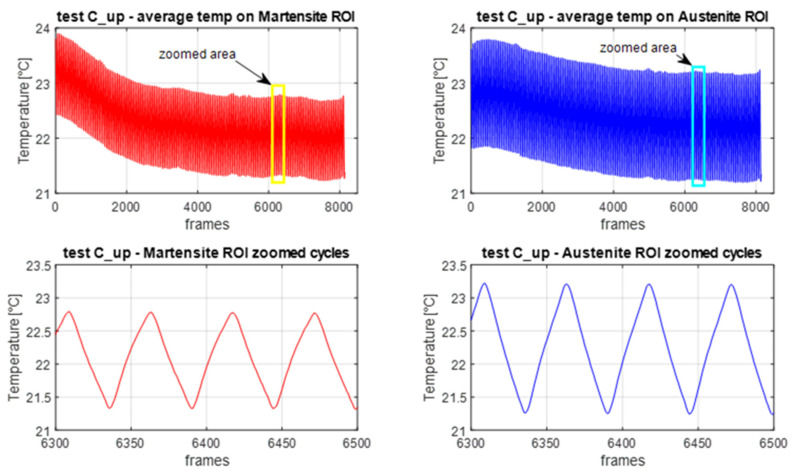
Plots of average temperature versus time during cycling from M and A ROIs for C_up cycles.

Figure 10 illustrates the mean temperature evolution in the A and M ROIs during C_up. An initial decay of the mean temperature was observed, which evolved towards a lower stable value, which was similar for both A and M ROIs. This drop is due to the dependence of temperature on the average stress. When the cyclic loading starts, the average stress goes from the upper plateau stress level to a lower stress level. The NiTi tested in this work, similar to what was observed by [19], exhibited a temperature change that has the same sign of the tensile stress change. Therefore, the drop of the average stress generates a decrease of temperature that is overlapped to the cyclic temperature change. Since the average stress remained fixed during cycling, the average temperature drop stabilised after a relatively short time. The average temperature evolution in the A ROI also has an initial increase. This is due to the last heat wave transferred by the A-M transformation front before this becomes fixed during cycling. This increase is not observed in the M ROI, likely because this is already warmer for the previous crossings of the transformation front.

Another feature of the temperature signal regards the low random noise and the good reproduction of a triangular wave, in response to the imposed triangular wave displacement modulation. This could well be the effect of a rather high thermoelastic coefficient *K*_1_ (see Equation (8)), as also reported in [19,20]. 

**Figure 11:**
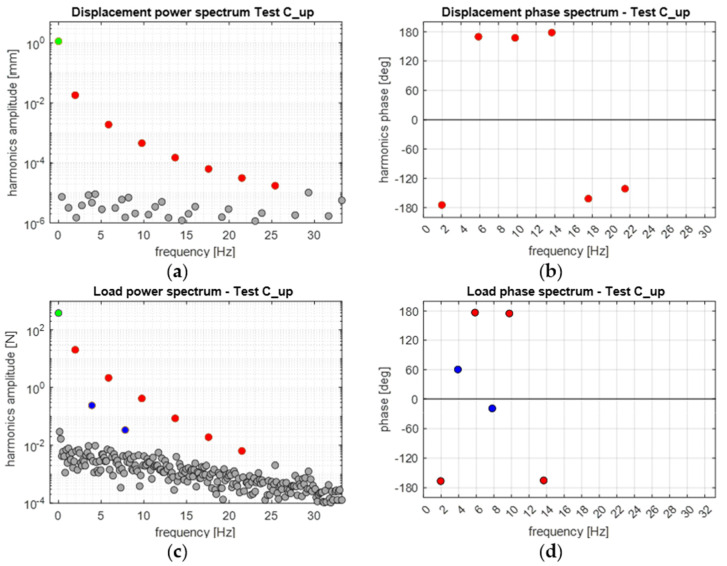
Displacement (**a**,**b**) and load (**c**,**d**) frequency spectra from applying the DFT to a windowed sequence during the C_up cycles: (**a**,**c**) amplitude and (**b**,**d**) phase spectra.

Figure 11 shows the amplitude and phase spectra, performed by the DFT, of the load and displacement signals acquired during the C_up cycling window. The DFT was performed on a sub-window which contained about 40 cycles, taken from the end of the cycling window, when the average temperature signal became stabilised. Moreover, the analysed signal included an optimised number of frames (see Section 3.5) such to reduce spectral leakage [31], and the signal sequence started from a wave trough (as in Figure 2), so that the phase of the harmonic at *ω* is ±180° (in accordance with the scheme in Figure 3).

The DFT spectra in Figure 11a,c show that the procedure to optimise the frequency bins against leakage was successful, and all significant harmonics were well distinguished over the noise bed. It is added here that by significant harmonic, we refer to a harmonic whose amplitude is noticeably above the noise bed. In particular, the displacement signal has significant harmonics only at odd multiples of *ω*, as expected from the triangular wave (see also the red dots in Figure 11c). Additionally, the phases of these odd harmonics oscillate around ±180°, as expected.

On the contrary, the load signal exhibits significant harmonics also at even multiples of *ω*. These might arise due to a non-linear response of the material within each cycle. It is noticed that the phase of the second and fourth harmonics seem to approach 0°, even if the second harmonic has a value of 60°. 

**Figure 12:**
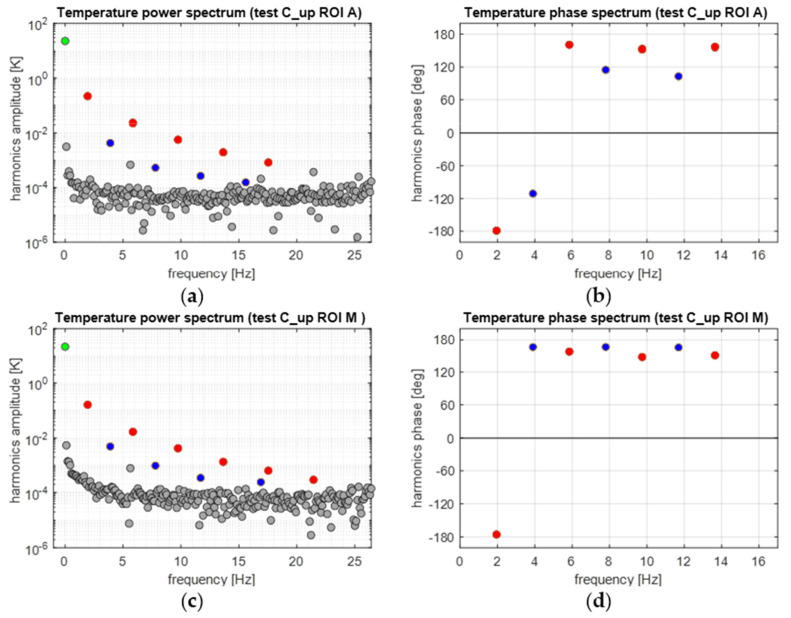
Temperature frequency spectra from applying the DFT to a windowed sequence during the C_up cycles: (**a**,**c**) amplitude and (**b**,**d**) phase spectra.

Figure 12 reports the equivalent DFT spectra obtained from the A ROI and M ROI average temperature sequences. Again, each sequence analysed considers the stabilised cycles, and starts from a wave trough. Figure 12a,c confirm that spectral leakage is effectively eliminated by implementing the procedure outlined in Section 3.5. The green dot indicates the average temperature at *ω* = 0°, while the red dots indicates harmonics at odd multiples of *ω* and blue dots at even multiples of *ω*. All other grey dots belong to the noise bed or are either generated by internal vibrations of the IR camera, or due to some residual spectral leakage.

It is highlighted here that while all odd harmonics (2*n* − 1)*ω* have a ±180° phase, in agreement with the triangular wave loading, The second and fourth harmonics follow different behaviours in the A and M ROIs. In the A ROI, the second harmonic seems to be opposite in phase to the second harmonic of the load (compare Figure 11b with Figure 12b). In the M ROI, all even harmonics have ±180° phases.

**Figure 13:**
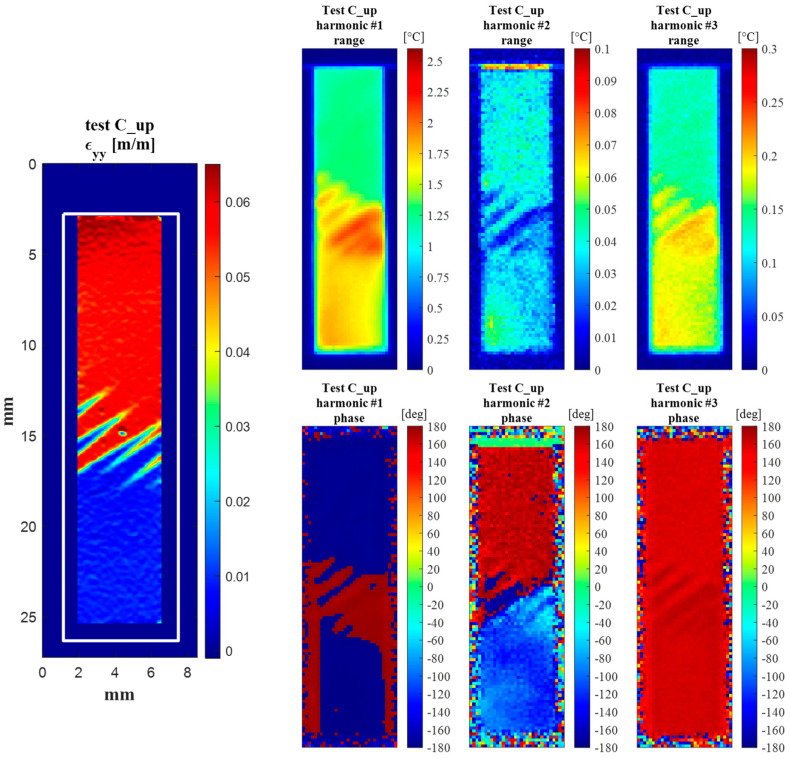
(**left**) DIC map of *ε_yy_* during the C_up cycling; (**right**) maps of the amplitude and phase of temperature harmonics at *ω*, 2*ω* and 3*ω*.

Finally, Figure 13 shows the maps of the amplitude and phase of the first, second and third harmonics, i.e., the harmonics at *ω*, 2*ω* and 3*ω*. A map of *ε_yy_* obtained from DIC is also shown to the side, showing the areas of the sample which have A or M phase and the frozen transformation fronts during the C_up cycling window. 

The temperature harmonics maps show that the behaviour observed from the average temperature signal from the A and M ROIs (see Figure 12) is similarly reproduced over all of the A and M regions. 

The harmonic amplitude maps at *ω* and 3*ω* have very similar features with the 3*ω* map, which seems just to be rescaled to lower values. This behaviour leads us to believe that the response of odd harmonics is proportional to the amplitude of the corresponding load harmonics. Furthermore, the amplitude of the *ω* harmonic is higher in the A region and lower in the M region. This result seems to be in accordance with results from [19,20], where the Austenite region exhibited a higher signal then the martensite. A direct evaluation of K^*^ = *K_o_* − *K*_1_*σ_m_* (see also Equation (8)) made in [19] indicates a higher |K^*^| for austenite, even though the comparison was made with the two A and M zones cycling at different average stresses, and then the contribution of *σ_m_* should also be considered. Average values taken from areas inside the Austenite and the Martensite zones indicate a ratio of thermoelastic signals of about 1.716/1.297. This ratio seems to be smaller than that indicated in [19], which might be explained by the average stress *σ_m_* being similar for the A and M regions tested in this work.

Another salient feature from Figure 13 is that the phase between the A and M regions is basically unchanged in the harmonics at *ω* and 3*ω*, while it changes significantly in the harmonic at 2*ω*. 

It is interesting also to point out that the sites of the transformation fronts exhibit higher harmonic amplitude at *ω* and 3*ω*, forming bands of higher signal having the same inclination as the Lüders bands. Additionally, the phase maps have a stripe-like signature over the transformation bands. The increase in amplitude signal is higher in the lower-right part of the transformation front zone, where the Lüders bands appear as thinner branches. This increase might be due to several reasons, which would require further investigation. First, it might indicate a local stress concentration, induced at the boundary between Austenite and Martensite material structure. It is also believed that the transverse deformations might change significantly, due to a change in the Poisson ratios of the Austenite and Martensite phases. 

The movement of the thin Lüders bands during cycling (due to the sample high staining) could also create a temperature modulation within the IR camera sensor pixel, which is instead fixed in space. This effect is well known in the TSA literature and is called pseudo-signal. Its correction requires the application of suitable motion compensation algorithms. Finally, another cause that can contribute to the localised non uniform signals is the lack of adiabaticity. The Lüders band front creates a local severe temperature gradient which might foster heat conduction at low loading frequencies. This last hypothesis might also explain the influence in the phase map, which indicates some localised phase shifting in the zones near the transformation fronts. In the rest of the sample, the phase signal is instead constant. 

#### 4.2.2. C_Down—Lower Plateau Cycling

Figure 14, Figure 15, Figure 16 and Figure 17 in this section report the same information as Figure 10, Figure 11, Figure 12 and Figure 13, but now for the C_down cycles.

**Figure 14:**
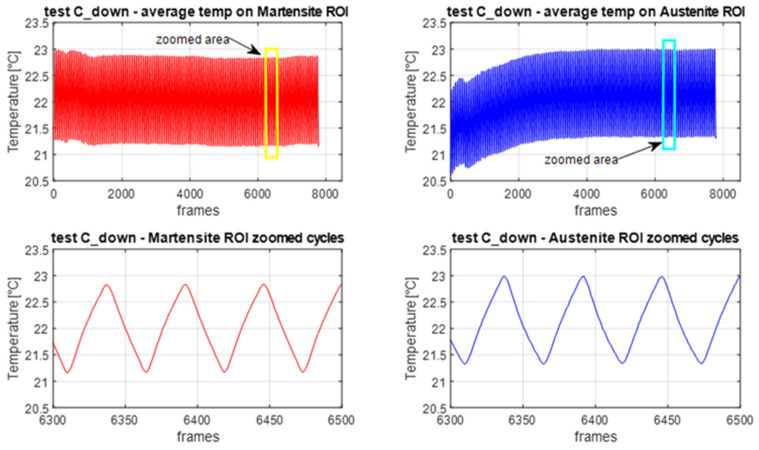
Plots of average temperature versus time during cycling from M and A ROIs for C_down cycles.

From Figure 14, it can be seen that the average temperature from the M ROI, this time, remains almost constant over the whole duration of cycling. In the A ROI, the average temperature initially increases, and then stabilises. Therefore, it seems that the Austenite phase is more sensitive to changes in average stress. 

**Figure 15:**
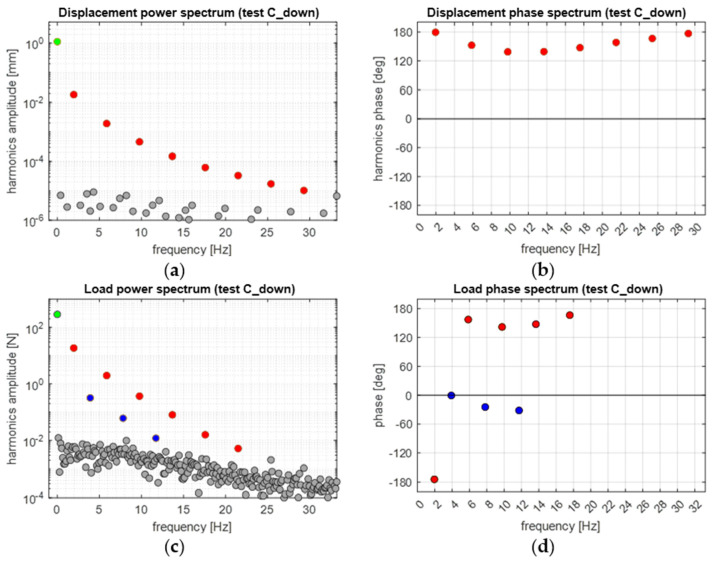
Displacement (**a**,**b**) and load (**c**,**d**) frequency spectra from applying the DFT to a windowed sequence during the C_down cycles: (**a**,**c**) amplitude and (**b**,**d**) phase spectra.

The displacement and load amplitude spectra in Figure 15a,c show similar features to those in Figure 11a,c. A noteworthy difference arises in the load phase spectra, where the second harmonic now has a phase value near 0°.

**Figure 16:**
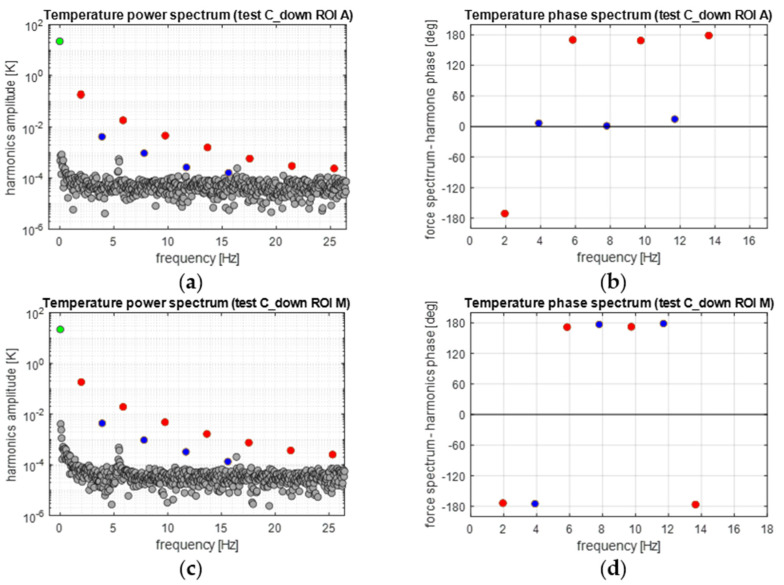
Temperature frequency spectra from applying the DFT to a windowed sequence during the C_down cycles: (**a**,**c**) amplitude and (**b**,**d**) phase spectra.

Figure 16 reports the temperature frequency spectra. One noteworthy difference with the previous C_up case is that the phase of the second harmonic at 2*ω* has a jump of 180° moving from the A to the M ROI. Comparing these values with those from Figure 15, it can be seen that for the harmonic at 2*ω*, the phases of the load and temperature are equal in the A ROI and opposite in the M ROI. This also seems to hold true for all other even harmonics (e.g., the fourth, the sixth, etc.). 

**Figure 17:**
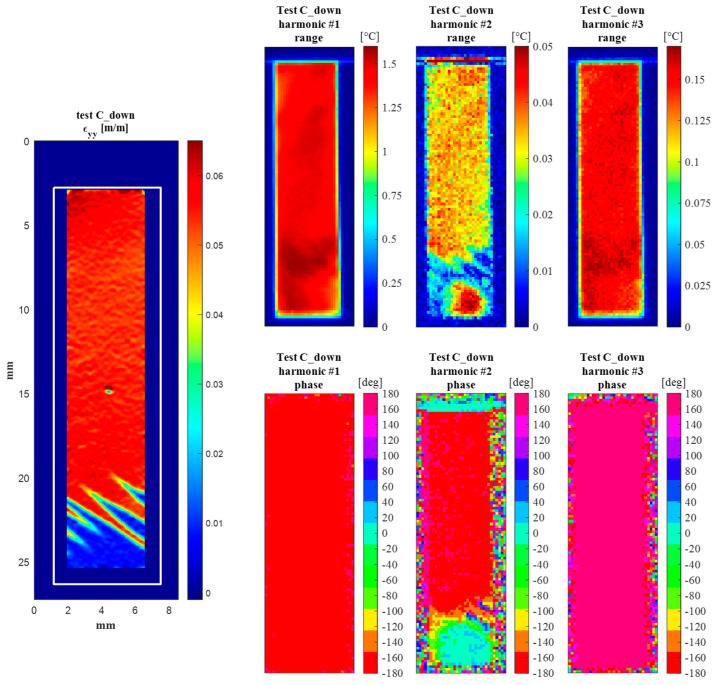
(**left**) DIC map of *ε_yy_* during the C_down cycling; (**right**) maps of the amplitude and phase of temperature harmonics at *ω*, 2*ω* and 3*ω*.

Finally, Figure 17 shows the full-field maps of the amplitude and phase of harmonics at *ω*, 2*ω* and 3*ω*. Again, the maps show that the behaviour observed from the average A and M ROIs is similarly reproduced over the whole A and M regions.

Similar comments can be made regarding the amplitude maps at *ω* and 3*ω*, with the 3*ω* map reproducing the same features at a lower scale. One noteworthy difference can instead be observed whereby the A and M regions now seem to have a similar amplitude. This result could be correlated with the fact that the Austenite region is now cycling at lower stresses (as can be seen in Figure 8c). It can also be observed that in the C_down test, the Austenite region is smaller and closer to the lower grip, while the martensite region covers a major area of the sample surface. 

The phase between the A and M regions remains substantially unchanged in the harmonics at *ω* and 3*ω*, while it has a 180° jump in the harmonic at 2*ω*, confirming what was seen in Figure 12b,d. 

## 5. Discussion of Results

**Figure 18:**
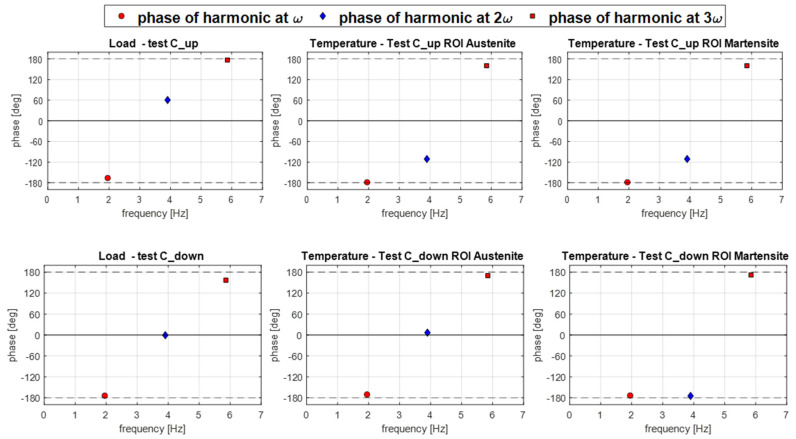
Phase responses of the three harmonics at *ω*, 2*ω* and 3*ω* in test C_up (**upper row**) and C_down (**lower row**), for the load signal and temperature signal averaged over the A ROI and M ROI.

In this section, the features of Figure 11, Figure 12 and Figure 13 and Figure 15, Figure 16 and Figure 17 are further commented on, discussing the correlation between the temperature modulation and the expected influence of the Thermoelastic Effect. To better follow the discussion, Figure 18 is proposed, showing the phase response of only the three harmonics at *ω*, 2*ω* and 3*ω*. 

It is undeniable that, under fatigue loading not involving an A⇄M transformation, i.e., under low-amplitude fatigue, the temperature is modulated by reversible thermomechanical heat sources. Moreover, this modulation seems to be strictly correlated with the load applied on the sample. It is also seen that an analysis of the harmonic response of temperature reveals several peculiar features which have the potential to identify the phase of the material (Austenite or Martensite) and the transformation fronts. 

It remains to be established whether the mentioned features can be fully explained within the theoretical framework of Thermoelastic Stress Analysis, or if this is insufficient to interpret or model the whole picture.

The resuls in this work show that the temperature harmonic at *ω*, 3*ω*, and distinctly all other significant harmonics at odd multiples of *ω* are always in phase with the corresponding displacement and load harmonics. This is so for both the Austenite and Martensite and at different mean stresses (e.g., for both C_up and C_down tests). This behaviour can only be justified by a second-order thermoelastic effect formulation expressed by the combination of Equation (8) and condition (9), i.e., when *dE/dT* is positive and higher than the threshold indicated in Equation (9). By adopting data from the literature (see Equation (10)), though, it should come out that the harmonic amplitude at 2*ω* should be of the same order of magnitude of the harmonic at *ω*. Results in this work instead found that the 2*ω* amplitude is at least two orders of magnitude smaller than the *ω* amplitude (see, e.g., Figure 12 or Figure 16). Furthermore, Equation (9) foresees that the harmonic at 2*ω* should be a cosine wave with 0° phase if the harmonic at *ω* is a cosine wave triggered (i.e., starting) at ±180° (see, e.g., Figure 3).

The outcome in terms of phases can be seen in Figure 18. In this work, Austenite regions have a 2*ω* phase that differs from 0° in the C_up test, and Martensite regions also have 2*ω* phases different from 0° and consistently equal to ±180°. 

In the C_up test, it was curiously observed that the harmonic at 2*ω* in the Austenite material is opposite in phase to the harmonic at 2*ω* of the load signal. This is a typical response expected from a first-order thermoelastic theory, which is in any case ruled out by the behaviour of the material at odd harmonics. Additionally, in the C_down test, the Austenite material had a phase value equal to the load, and at 0° as predicted by Equation (9). 

The Martensite 2*ω* phase is instead always against the prediction of the second-order thermoelastic law (Equation (9)) and compliant with the first-order thermoelastic theory. Its 2*ω* harmonic had a ±180° phase shift at both C_up and C_down.

In general, the DFT on the load signal saw that the load had a significant second harmonic component at 2*ω*, which was not present in the machine-controlled displacement signal. This load component at 2*ω* likely arises from a non-linear material response.

In conclusion, there seems to be enough evidence that the Thermoelastic Effect theory and the thermoelastic heat source are not able to fully justify the amplitude and phase of the significant harmonics measured from the Austenite and Martensite regions. 

As recalled in the introduction, some authors have tried to investigate the influence on the thermoelastic behaviour of an intermediate R-Type transformation between Austenite and Martensite. In [23], it was shown that an A→R→M PT can be stress activated, leading to a reduction in the Young’s modulus before reaching the upper plateau of the M PT.

Here it can be observed that an intermediate R-Type transformation can also act as a further reversible thermomechanical heat source, due to the elastocaloric effect associated with this transformation. Some authors have investigated the thermomechanical behaviour of a A⇄R PT [34,35,36]. It is reported that this transformation occurs homogeneously over the sample gauge area, unlike the Lüders-like behaviour of the A⇄M PT. Furthermore, a smaller reversible elastocaloric effect is present which produces a temperature increase in the loading A⇄R path, and a temperature decrease during the unloading R⇄A path. This is also in agreement with the observed in-phase relation between the load and temperature.

As this R-Type intermediate PT seems to deploy its effects in a reversible way and in a uniform and gradual manner, its effect can be modelled via the heat diffusion equation (see Equation (1)) and added as a thermomechanical heat source in the right-hand side of Equation (1). If all other thermomechanical heat sources are neglected and the temperature change occurs under adiabatic conditions, then Equation (1) may be rewritten as:

(15) $$\rho C_{\varepsilon }\dot{T}=T\frac{\partial A_{x}}{\partial T}\dot{x}$$

where $A_{x}=\rho \partial H/\partial x$ is the thermodynamic force associated with the internal variable *x*, which describes the material PT of the R-type [26]. It can be observed that Equation (15) is formally similar to Equation (2). Therefore, if an early A⇄R PT is present in the range of low-amplitude cycling loading, and it is assumed that $\partial A_{x}/\partial T$ is positive, as the experimental evidence seems to suggest, then a relation formally similar to Equation (7) would be obtained as:

(16) $$\Delta T=\frac{T_{o}}{\rho C_{\varepsilon }}\frac{\partial A_{x}}{\partial T}\Delta x=K_{3}\Delta x$$

where the temperature change is correlated with the amount of R-phase transformation Δ*x* via a positive *K*_3_ coefficient. If the R-Type transformation level, Δ*x*, is linearly correlated with the load, then the temperature will still be linearly correlated with the load and will follow the same waveform, as it seems to happen in NiTi under low-amplitude fatigue.

To validate the above assumptions, summarised in Equation (16), further specific tests will be required, which goes beyond the original purposes of this work. Future work is, however, being planned to also explore the role of intermediate R-Type transformations and their influence on the thermoelastic response of the material. 

## 6. Conclusions

A tensile NiTi sample exhibiting a super-elastic behaviour at room temperature was tested under cyclic loading, applied via displacement controlled triangular wave. The applied displacements made it possible to introduce different macro regions of Austenite and Martensite, while the applied cyclic displacement ranges were sufficiently small to avoid further PT and freeze the existing transformation fronts. 

Digital Image Correlation was used, making it possible to map the Austenite and Martensite regions, while temperature sampling with an IR camera made it possible to investigate the peculiar temperature modulation. 

The frequency domain analysis proposed in this work was demonstrated to be highly informative, as the maps of amplitude and phase of the significant harmonics of temperature carry a signature of the material phase status and of the PT fronts. Future work will investigate how such features are reproduced when a bidimensional stress field is introduced by means of a stress raiser (e.g., holes, notches, cracks, etc.). 

The temperature harmonics at the main loading frequency *ω* and at odd and even multiples of *ω* were obtained and compared against the theoretical predictions made via the first- and second-order theories of the Thermoelastic Effect. Results show that the measured temperature has a periodic modulation whose waveform seems to correlate well with the waveform of the applied load. Furthermore, the amplitude and phase maps of the harmonics at *ω* and 3*ω* show similar features, with the 3*ω* output just rescaled to lower values. The phase response at 2*ω* is instead different between the austenite and the martensite regions, and generally not compliant with the predictions of the second-order theory of the Thermoelastic Effect.

Further work is therefore needed to better characterise the nature of the reversible heat sources that determine the modulation of temperature under cyclic low-amplitude loadings of NiTi alloys. Future investigations by the authors will be specifically focused on the analysis of intermediate R-Type PTs, since various experimental and theoretical results show that this could play a significant role as a potential reversible thermomechanical heat source. 

## Figures and Tables

**Table 1:** Denomination of the tests loading profiles.

Type of Experiment	Test Denomination
Quasi-static	QS
Cyclic no A⇄M PT from upper plateau	C_up
Cyclic no A⇄M PT from lower plateau	C_down

## Data Availability

The data presented in this study are available on request from the corresponding author.
